# Efficacy and safety of acalabrutinib with best supportive care versus best supportive care in patients with COVID-19 requiring hospitalization

**DOI:** 10.1093/immhor/vlaf023

**Published:** 2025-06-03

**Authors:** Phillip Scheinberg, Matt R Khoshnevis, Philip A Robinson, Alfredo Guerreros, Victor A H Sato, Benedito A L Fonseca, Hans W Prozesky, José Omar Chacón Romero, Laura Fogliatto, Barry R Meisenberg, David J Park, Ashok Gupta, Priti Patel, Danielle M Townsley, Lianqing Zheng, Veerendra Munugalavadla

**Affiliations:** Division of Hematology, Hospital A Beneficência Portuguesa, São Paulo, Brazil; Center for Critical Care, Houston Methodist Hospital, Houston, TX, United States; Infection Prevention and Hospital Epidemiology, Hoag Memorial Hospital Presbyterian, Newport Beach, CA, United States; Department of Medicine, Clinica Internacional, Lima, Peru; International Research Center, Hospital Alemao Oswaldo Cruz, São Paulo, Brazil; Hospital das Clínicas da Faculdade de Medicina de Ribeirão Preto da Universidade de São Paulo (FMRP), São Paulo, Brazil; Division of Infectious Diseases, Department of Medicine, Stellenbosch University and TREAD Research, Tygerberg Hospital, Cape Town, South Africa; Hospital Sub-direction, IMSS HGR No. 1 Dr Carlos Mac Gregor Sanchez Navarro, Mexico City, Mexico; Hematology and Hemotherapy Department, Hospital de Clinicas de Porto Alegre, Porto Alegre, Brazil; Department of Medicine, Anne Arundel Medical Center, Annapolis, MD, United States; Hematology and Oncology, Providence St Jude Medical Center/Providence Medical Foundation, Fullerton, CA, United States; Oncology Research and Development, AstraZeneca, Gaithersburg, MD, United States; Haematology Research and Development, AstraZeneca, South San Francisco, CA, United States; Oncology Research and Development, AstraZeneca, Gaithersburg, MD, United States; Oncology Biometrics, AstraZeneca, South San Francisco, CA, United States; Haematology Research and Development, AstraZeneca, South San Francisco, CA, United States

**Keywords:** acalabrutinib, best supportive care, bruton tyrosine kinase inhibitor, COVID-19, hospitalized patients

## Abstract

The efficacy and safety of acalabrutinib, a Bruton tyrosine kinase (BTK) inhibitor, was evaluated in 2 phase 2 studies in hospitalized patients with coronavirus disease 2019 (COVID-19) who received acalabrutinib + best supportive care (BSC) versus BSC alone (Clinicaltrials.gov: NCT04380688 and NCT04346199). The primary endpoint was the percentage of patients alive and free of respiratory failure on day 14 (rest of the world [RoW] study) and day 28 (US study). In the RoW study, 177 patients were randomized (acalabrutinib + BSC: n = 89; BSC: n = 88); in the US study, 62 patients were randomized (acalabrutinib + BSC: n = 31; BSC: n = 31). The percentage of patients who met the primary endpoint was similar in both studies (RoW study: acalabrutinib + BSC: 83.1%, BSC: 90.9%; US study: acalabrutinib + BSC: 80.6%, BSC: 83.9%). No new safety concerns were reported. Overall, no significant clinical benefit of adding acalabrutinib to BSC in patients hospitalized with COVID-19 was observed.

## Introduction

Infection with coronavirus disease 2019 (COVID-19), also known as severe acute respiratory syndrome coronavirus 2 (SARS-CoV-2) is associated with symptoms of fever, cough, fatigue, and shortness of breath.^1–4^ In hospitalized patients with COVID-19, chest computed tomography (CT) imaging frequently reveals ground-glass opacity in the lungs and patchy bilateral shadowing.^1–4^ As of March 2024, the World Health Organization (WHO) reported more than 775 million confirmed cases of COVID-19 and over 7 million deaths globally.^5^ Approximately 81% of COVID-19 cases are asymptomatic or mild, 14% are severe (requiring ventilation in an intensive care unit), and 5% of patients have critical illness that includes respiratory failure, septic shock, and/or multiple organ dysfunction.^2^ The risk of mortality increases with age, male sex, and the presence of comorbidities, such as cardiovascular disease, diabetes, and chronic obstructive pulmonary disease or asthma, with reported death rates of up to 15% of COVID-19 cases.^2^^,^^6^^,^^7^

Acute respiratory distress syndrome (ARDS) in patients with COVID-19 is associated with a severe inflammatory response (also referred to as a “cytokine storm”), which is associated with elevated serum levels of multiple inflammatory cytokines and chemokines (eg, interleukin-1 beta [IL-1β], IL-6, IL-7, IL-8, IL-9, IL-10, granulocyte colony-stimulating factor, granulocyte-macrophage colony-stimulating factor, interferon-gamma [IFN-γ], IFN-γ-induced protein 10 kDa, monocyte chemoattractant protein-1 [MCP-1], and macrophage inflammatory protein-1 alpha [MIP-1α]).^3^^,^^8^

The cytokine storm response observed in patients with COVID-19 has characteristics that overlap with macrophage activation syndrome, suggesting that the innate immune system may represent an effective target for treatment.^8^^,^^9^ Corticosteroids cause immune suppression by impairing the innate immune system.^10^ Therefore, by modulating inflammation-mediated lung injury, corticosteroids may reduce the progression to respiratory failure and death in patients with COVID-19.^11^ Intervention with corticosteroids has been shown to decrease the mortality of hospitalized patients with COVID-19 who required oxygen; however, in patients who did not require oxygen, steroid use was associated with an increased risk for disease progression.^12^ In the Randomized Evaluation of COVID-19 Therapy (RECOVERY) study, among hospitalized patients with COVID-19 receiving the usual standard of care (ie, mechanical ventilation or supplemental oxygen), the addition of 6 mg of dexamethasone administered once daily for up to 10 days led to a reduced 28-d mortality rate relative to patients who received invasive mechanical ventilation alone.^11^ However, there was no evidence to support the use of dexamethasone in patients who did not receive respiratory support.^11^ Further, among patients who received oxygen, dexamethasone treatment was associated with both a lower risk of needing invasive mechanical ventilation, and an increased likelihood of extubation among those already receiving mechanical ventilation.^11^ In both groups, dexamethasone increased the potential for patients to be alive and discharged on day 28.^11^ Currently, the Medicines and Healthcare products Regulatory Agency in the United Kingdom,^13^ the WHO,^14^ and the National Institutes of Health^15^ recommend the use of corticosteroids in hospitalized patients with COVID-19 who require supplemental oxygen, regardless of mechanical ventilation support. Steroid use is not recommended in patients with asymptomatic or milder forms of disease.^12^

Bruton tyrosine kinase (BTK) inhibitors are candidate therapies for COVID-19 as BTK is a critical mediator of B-cell receptor signaling and adaptive immunity and plays a key role in numerous immunologic signaling networks associated with innate immunity (eg, toll-like receptor [TLR] signaling).^16^ TLR-dependent BTK-activation promotes nuclear factor kappa B– and IFN-regulatory factor–dependent transcription of inflammatory cytokines and interferons.^16^ Further, overexpression of BTK in the lungs is associated with development of ARDS and lung injury.^17^ Increased BTK activity also has been reported in CD14+ monocytes from patients with severe COVID-19 compared with those from healthy volunteers, with similar total BTK levels.^8^ Consequently, BTK was hypothesized to be a relevant inhibition target to reduce hyperinflammation and prevent severe lung injury in patients with COVID-19.^18^

In a preliminary, non-controlled clinical study of 19 patients hospitalized with severe COVID-19, off-label use of the selective BTK inhibitor acalabrutinib improved oxygenation in the majority of patients, often within 1 to 3 d, with no discernible toxicity.^8^ Acalabrutinib administration to patients receiving supplemental oxygen led to an 82% improvement in oxygenation. However, the benefit to patients on ventilators was less dramatic, with only half of the patients being extubated.^8^ These preliminary improvements in oxygenation and good tolerability with acalabrutinib treatment in this small number of patients suggested that more extensive evaluation of acalabrutinib in a larger cohort of patients with COVID-19 hospitalization was needed.

This article reports the efficacy, safety, and exploratory biomarker analysis from 2 randomized phase 2 studies in patients with COVID-19 who received acalabrutinib + best supportive care (BSC) versus BSC alone. These studies conducted during the early period of the COVID-19 pandemic in 2020 included a larger number of patients (total of 239 randomized across both studies) compared with the prior study to comprehensively assess the potential benefits of acalabrutinib treatment in hospitalized patients with COVID-19 receiving BSC.

## Materials and methods

### Study design and population

The CALAVI phase 2 studies were multicenter, randomized, open-label studies evaluating the efficacy and safety of acalabrutinib plus BSC versus BSC alone in patients hospitalized with COVID-19 in the United States and the rest of the world (RoW); separate studies were a result of guidance from regulatory agencies at the start of the COVID-19 pandemic. The studies were conducted between June 2020 and November 2020. Inclusion criteria included patients aged ≥18 y; the presence of SARS-CoV-2 confirmed using WHO criteria (including positive nucleic acid test of any specimen [eg, respiratory, blood, urine, stool, or other bodily fluid]) within 7 d of randomization; and radiographically documented COVID-19 pneumonia requiring hospitalization and oxygen saturation (SpO_2_) of <94% on room air, or a requirement for supplemental oxygen. Patients with known medical resuscitation within 14 d of randomization, suspected uncontrolled infection, and uncontrolled or untreated symptomatic arrhythmias, myocardial infarction within the last 6 wk, or congestive heart failure (New York Heart Association class 3 or 4) were excluded.

Studies were conducted per the ethical principles in the Declaration of Helsinki and are consistent with the International Conference on Harmonisation and Good Clinical Practice guidelines and applicable regulatory requirements. The clinical studies, including protocols and patient-informed consent documents, were approved by an institutional review board or independent ethics committee at each study site before study initiation. All patients provided written informed consent before study participation. Both studies are registered with ClinicalTrials.gov, NCT04380688 (US) and NCT04346199 (RoW).

### Randomization and treatment

**Figure 1.**
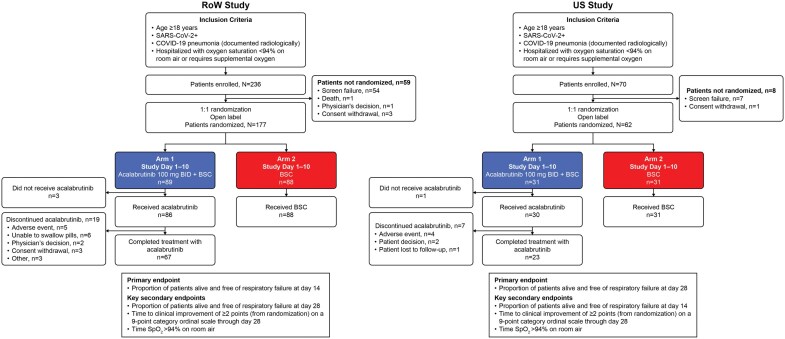
Study design, patient disposition, and endpoints. BID, twice daily; BSC, best supportive care; COVID-19, coronavirus disease 2019; RoW, rest of the world; SARS-CoV-2+, severe acute respiratory syndrome coronavirus 2 positive; SpO_2_, oxygen saturation; US, United States.

Patients were randomized (1:1) to receive oral acalabrutinib 100 mg twice daily (BID) for 10 d plus BSC (arm 1) or BSC alone (arm 2) (Fig. 1). Randomization was stratified according to the following prognostic factors for poor outcome: age (≥65 vs <65 yr), absence or presence of ≥1 of the following comorbidities: cardiovascular disease (defined as either heart failure of New York Heart Association class ≥2 or hypertension requiring treatment), diabetes mellitus requiring treatment, chronic obstructive pulmonary disease or asthma requiring treatment, presence of an active solid tumor, or hematological malignancy.

Acalabrutinib treatment was administered for 10 d or a maximum of 20 doses. The type of BSC used was at the investigator’s discretion and was per institutional guidelines. The following therapies were not allowed in this study: treatment with a strong cytochrome P450 3A (CYP3A) inhibitor within 7 d before the first dose of study drug, or a CYP3A inducer within 14 d before the first dose of study drug; prior immunomodulatory/immunosuppressive treatment intended as a specific treatment for COVID-19, but not considered standard of care according to institutional guidelines; anticoagulation with warfarin or equivalent vitamin K antagonists within 7 d of the first dose of acalabrutinib; and dual antiplatelet and therapeutic anticoagulant therapy (US study only). The use of therapies such as remdesivir, therapeutic plasma, corticosteroids, or other immunomodulatory agents was permitted if recommended by local authorities and was part of institutional policies or guidelines.

### Endpoints

The primary efficacy endpoint was the proportion of patients alive and free of respiratory failure at day 28 (US study) or day 14 (RoW study). Different endpoints for the US and the RoW were informed by guidance from regulatory agencies at the start of the pandemic. Respiratory failure was defined based on the use of any of the following modalities: endotracheal intubation and mechanical ventilation; oxygen delivered by high-flow nasal cannula; noninvasive positive pressure ventilation or continuous positive airway pressure; or extracorporeal membrane oxygenation.

Key secondary efficacy endpoints included the proportion of patients alive and free of respiratory failure at day 14 (US study) or day 28 (RoW study); time to clinical improvement of ≥2 points (from randomization) on a 9-point category ordinal scale through day 28 (0: uninfected, no clinical or virological evidence of infection; (1) ambulatory, no limitation of activities; (2) ambulatory, limitation of activities; (3) hospitalized—mild disease, no oxygen therapy; (4) hospitalized—mild disease, oxygen by mask or nasal prongs; (5) hospitalized—severe disease, noninvasive ventilation or high flow oxygen; (6) hospitalized—severe disease, intubation and mechanical ventilation; (7) hospitalized—severe disease, ventilation and additional organ support [eg, vasopressors, renal replacement therapy, extracorporeal membrane oxygenation]; (8) death; if a patient did not demonstrate a clinical improvement of ≥2 points on or before day 28 and did not withdraw or become lost to follow-up from the study prior to day 28, the data were censored at day 28; if a patient did not have an event but withdrew from the study, or was lost to follow-up prior to day 28, the data were censored at the last known date when the patient had not demonstrated a clinical improvement of ≥2 points up to day 28); and time to SpO_2_ of >94% on room air.

Safety endpoints included any adverse event (AE), including type, frequency, severity, and relationship to study treatment, serious AEs (SAEs), AEs leading to study drug discontinuation, and laboratory test abnormalities. Exploratory endpoints included change from baseline of the cytokine/chemokine profile, of the SARS-CoV-2 viral load and antibody levels, and of the BTK occupancy. Correlative analysis among treatment effects, biomarkers, and study drug exposure was also explored.

### Study assessments

On-study assessments included vital signs; concomitant medications; clinical improvement score on a 9-point ordinal scale (from 0 [uninfected, no clinical or virological evidence of infection] to 8 [death]); AEs; and survival status. Routine laboratory assessments (hematology, serum/plasma chemistry, arterial blood gases, fibrinogen, prothrombin time, activated partial thromboplastin time, international normalized ratio, D-dimer, C-reactive protein [CRP], cardiac troponin 1, procalcitonin, serum ferritin, hepatitis B and C testing, urine or serum pregnancy tests), chest imaging, electrocardiogram, and echocardiogram (as clinically indicated) as well as central laboratory assessments of correlative samples, immunophenotyping, assessment of SARS-CoV-2 viral load/viral shedding, and SARS-CoV-2 quantitative serology were also performed.

### Biomarker analysis

Viral load (copies/ml) was determined by real-time quantitative polymerase chain reaction (RT-qPCR; Viracor) from nasopharyngeal swabs. The imputation method used for viral load was 714 copies/ml; therefore, when test results were labeled as “detected <714,” they were imputed as 71,000,000 copies/ml. Serum immunoglobulin G antibody levels against SARS-CoV-2 nucleocapsid, SARS-CoV-2 receptor-binding domain (RBD), and SARS-CoV-2-spike 1 proteins were measured quantitatively via Meso Scale Discovery (MSD) from baseline through day 28 and are presented by subgroup and visit. The imputation method for serology was that if the test results were lower than the lower limit of detection (LLOD) × the dilution factor, then the final results were imputed as 100 (dilution factor) × the LLOD; if the test results were greater than the upper limit of detection (ULOD) × the dilution factor, then the final results were imputed as 5000 × the ULOD. Qualitative strain-specific serostatus was determined using the Platelia SARS-CoV-2 total anti-nucleocapsid antibody test (Biodesix) at screening. Serostatus was defined as: ≥1.0, positive; <0.8, negative; and 0.8< × <1.0 equivocal. Plasma was tested using the MSD method for biomarkers including acute phase proteins, cytokines, and chemokines. For cytokines and chemokines, the imputation method was that if the test results were below the lower limit of quantification (LLOQ) × the dilution factor, then the final results were imputed as LLOQ × the dilution factor; if the test results were above the upper limit of quantification (ULOQ) × the dilution factor, then the final results were imputed as ULOQ × the dilution factor.

### Statistical analysis

The total numbers of patients planned for the US and RoW studies were 60 and 140, respectively. Based on published literature reporting death rates and the need for intensive care unit admission in patients hospitalized for COVID-19,^4^^,^^19–22^ it was assumed that the proportion of patients treated with BSC who were alive and free of respiratory failure at days 14 and 28 would be 70%.

The primary objective of the US study was to evaluate the safety and preliminary efficacy of acalabrutinib plus BSC versus BSC. A sample size of 60 for the US study would provide the half-width of the 2-sided 90% confidence interval (CI) for the observed treatment difference to be 16.4% using an unpooled estimate for variance. The US study had 64% power, with a 2-sided type 1 error of 0.1, to detect a difference of 20% between groups (ie, 70% for BSC and 90% for acalabrutinib + BSC). A sample size of 140 for the RoW study allowed for ≥85% power, with a 2-sided type 1 error of 0.05, to detect a difference of 20% between groups for the primary endpoint.

The analysis populations were defined as follows: the full analysis set (FAS) included all patients who were randomized on an intent-to-treat (ITT) basis, with treatment arms compared according to the randomized study treatment, regardless of the treatment received. The per-protocol (PP) analysis set included only patients without important detected protocol deviations which could affect the efficacy endpoints. Comparisons using this analysis set were performed according to the actual treatment received first. Comparisons using the safety analysis set (safety population) were based on the treatment patients received; data from patients who received ≥1 dose of acalabrutinib were included in the acalabrutinib + BSC groups, while data from patients who were randomized to acalabrutinib + BSC arm but who did not receive any acalabrutinib were included in the BSC group.

For the primary efficacy endpoint, point estimates and the associated 90% CIs (for the US study) and 95% CIs (for the RoW study) were calculated for each treatment arm. The Cochran-Mantel-Haenszel χ2 test, both unstratified and stratified by age (≥65 vs <65 yr) and comorbidities (present vs absent), was used to compare the primary endpoint between the 2 treatment arms. For the continuous secondary endpoints, summary statistics (n, mean, median, standard deviation [SD], minimum, and maximum) are presented by treatment arm. Time-based secondary endpoints were analyzed using the Kaplan-Meier method, with hazard ratios (HRs) and corresponding 90% CIs (for the US study) and 95% CIs (for the RoW study) estimated using Cox proportional-hazards models, stratified by randomization stratification factors. Safety endpoints and assessments are presented using summary statistics. AEs are described using version 23.0 of the Medical Dictionary for Regulatory Activities (MedDRA) and were graded using the Common Terminology Criteria for AEs (CTCAE) version 5.0. The frequency of AEs was summarized by system organ class and preferred term, as per MedDRA, and by the worst reported National Cancer Institute CTCAE grade. AEs in the acalabrutinib + BSC arm were defined as events occurring from the start date up to the last dose of study treatment plus 28 (±3) d. For the BSC arm, AEs were defined as events occurring from the start date up to 38 (±3) d from randomization. No imputation of values for missing clinical data was performed, except for missing or partial start and end dates for AEs and concomitant medications, which were imputed per prespecified, conservative imputation rules.

For all biomarker panels (ie, viral load, serology, immunophenotyping, and cytokine/chemokine), continuous data were summarized with the use of descriptive statistics (number of observations, mean, SD, median, first quartile, third quartile, minimum, and maximum). Frequencies and percentages were used to summarize categorical (discrete) data by treatment and by subgroup. Boxplots were utilized (in either linear scale or semi-log scale) to compare the biomarker levels at each visit by treatment.

## Results

### Patient population and study disposition

In the RoW study, 236 patients were enrolled and 177 patients were randomized (acalabrutinib + BSC, n = 89; BSC alone, n = 88). A total of 19 (22.1%) patients in the acalabrutinib + BSC arm discontinued treatment with acalabrutinib. The most common reasons for discontinuation of study treatment were an inability to swallow pills (6/19, 31.6%) and AEs (5/19, 26.3%) (Fig. 1). In the US study, 70 patients were enrolled and 62 patients were randomized (acalabrutinib + BSC, n = 31; BSC alone, n = 31). A total of 7 (23.3%) patients in the acalabrutinib + BSC arm discontinued treatment with acalabrutinib. The most common reason for discontinuation of study treatment was AEs (4/7, 57.1%).

In both studies, the majority of patients were <65 yr of age and male (Table 1). Most patients had 0 or 1 comorbidity, with the most common being cardiovascular disease and diabetes requiring treatment. There was a higher proportion of patients with respiratory failure in the US study, and these patients were more severely ill at baseline compared with those in the RoW study. There was a higher proportion of patients with a body mass index (BMI) of ≥30 kg/m^2^ at baseline in the US study (20/31 [64.5%] patients in the acalabrutinib + BSC arm and 18/31 [58.1%] patients in the BSC-alone arm) compared with the RoW study (31/89 [34.8%] patients in the acalabrutinib + BSC arm and 36/80 [45.0%] patients in the BSC-alone arm). Almost all patients in both studies (>96%) had an SpO_2_ of <95%. The number of patients on oxygen treatment, defined as utilization of either nasal cannula, mask, or high-flow nasal cannula, for the acalabrutinib + BSC and BSC-alone arms was 28/30 (93.3%) and 27/32 (84.4%), respectively, for the US study and 77/86 (89.5%) and 82/91 (90.1%), respectively, in the RoW study at baseline. At day 28, the number of patients on oxygen treatment in the acalabrutinib + BSC and BSC-alone arms was 3/26 (11.5%) and 4/28 (14.3%), respectively, for the US study and 1/76 (1.3%) and 1/81 (1.2%), respectively, for the RoW study.

**Table 1.** Patient characteristics and baseline demographics.

Characteristics	RoW study		US study	
	ACA + BSC (n = 89)	BSC alone (n = 88)	ACA + BSC (n = 31)	BSC alone (n = 31)
**<65 years of age**	61 (68.5)	60 (68.2)	26 (83.9)	25 (80.6)
**Sex**				
Male	60 (67.4)	64 (72.7)	18 (58.1)	22 (71.0)
Female	29 (32.6)	24 (27.3)	13 (41.9)	9 (29.0)
**BMI ≥30 kg/m^2^**	31 (34.8)	36/80 (45.0)	20 (64.5)	18 (58.1)
**Number of comorbidities per patient** ^a^				
0	40 (44.9)	39 (44.3)	11 (35.5)	10 (32.3)
1	25 (28.1)	34 (38.6)	11 (35.5)	13 (41.9)
2	23 (25.8)	15 (17.0)	7 (22.6)	6 (19.4)
3	1 (1.1)	0	2 (6.5)	1 (3.2)
4	0	0	0	1 (3.2)
**Type of comorbidity**				
CVD	41 (46.1)	33 (37.5)	13 (41.9)	12 (38.7)
DM requiring treatment	29 (32.6)	25 (28.4)	13 (41.9)	12 (38.7)
COPD or asthma requiring treatment	2 (2.2)	6 (6.8)	5 (16.1)	7 (22.6)
Current active malignancy	2 (2.2)	0	0	1 (3.2)
**Patients without an SpO_2_ of >94%**	86 (96.6)	87 (98.9)	30 (96.8)	31 (100)
**Respiratory failure**	5 (5.6)	3 (3.4)	1 (3.2)	6 (19.4)
Endotracheal intubation and MV	2 (2.2)	1 (1.1)	0	1 (3.2)
HFNC	2 (2.2)	0	1 (3.2)	3 (9.7)
NIPPV or CPAP	1 (1.1)	2 (2.3)	0	2 (6.5)
ECMO	0	0	0	0
**Duration of hospitalization prior to randomization, median (range), days**	3.0 (0–17)	3.0 (0–14)	2.0 (1–5)	3.0 (1–10)
**Hematology**				
Neutrophils^b^ (10^9^/l), mean (SD)	8.0 (4.1)	6.8 (3.1)	7.1 (4.3)	7.0 (3.2)
Platelets (10^9^/l), mean (SD)	288.1 (112.0)	273.5 (100.2)	249.2 (76.6)	271.6 (78.4)
**Mean (SD) lymphocyte counts** ^b^ **, 10^9^/l**	1.17 (0.58)	1.14 (0.66)	0.80 (0.42)	1.07 (0.61)

Data are presented as n (%) unless otherwise noted.

a BMI was not assessed as a comorbidity.

b Number of patients assessed for the neutrophil and lymphocyte counts at baseline were ACA + BSC (n = 84) and BSC (n = 86) for the RoW study, and ACA + BSC (n = 19) and BSC (n = 22) for the US study.

ACA, acalabrutinib; BMI, body mass index; BSC, best supportive care; COPD, chronic obstructive pulmonary disease; CPAP, continuous positive airway pressure; CVD, cardiovascular disease; DM, diabetes mellitus; ECMO, extracorporeal membrane oxygenation; HFNC, high-flow nasal cannula; MV, mechanical ventilation; NIPPV, non-invasive positive pressure ventilation; RoW, rest of the world; SD, standard deviation; SpO_2_, oxygen saturation.

The most common types of BSC received by patients in both studies are summarized in Table 2. There was a high use of corticosteroids in both studies (>85% across all groups), including dexamethasone, hydrocortisone, methylprednisolone, prednisolone, and prednisone, as well as the antiviral drug remdesivir in the US study (US study: acalabrutinib + BSC: 77.4% vs BSC alone: 58.1%; RoW study: acalabrutinib + BSC: 14.6% vs BSC alone: 8.0%).

**Table 2.** Best supportive care administered on study: steroids, interleukin-6 agents, and antivirals (full analysis set).

Best supportive care administered on study	RoW study		US study	
	ACA + BSC (n = 89)	BSC alone (n = 88)	ACA + BSC (n = 31)	BSC alone (n = 31)
**Glucocorticoids**	**76 (85.4)**	**77 (87.5)**	**30 (96.8)**	**29 (93.5)**
Dexamethasone	49 (55.1)	54 (61.4)	27 (87.1)	28 (90.3)
Dexamethasone sodium phosphate	0	1 (1.1)	0	0
Hydrocortisone	0	2 (2.3)	1 (3.2)	0
Hydrocortisone sodium succinate	0	1 (1.1)	2 (6.5)	2 (6.5)
Methylprednisolone	28 (31.5)	18 (20.5)	2 (6.5)	3 (9.7)
Methylprednisolone sodium succinate	16 (18.0)	8 (9.1)	1 (3.2)	1 (3.2)
Prednisolone	4 (4.5)	0	0	0
Prednisone	4 (4.5)	10 (11.4)	5 (16.1)	1 (3.2)
**Interleukin inhibitors**	**3 (3.4)**	**5 (5.7)**	**1 (3.2)**	**1 (3.2)**
Tocilizumab	3 (3.4)	5 (5.7)	1 (3.2)	1 (3.2)
**Antivirals**				
**Nucleosides and nucleotides excluding reverse transcriptase inhibitors**	13 (14.6)	7 (8.0)	24 (77.4)	18 (58.1)
Remdesivir	13 (14.6)	7 (8.0)	24 (77.4)	18 (58.1)
**Neuraminidase inhibitors**	3 (3.4)	3 (3.4)	0	0
Oseltamivir	3 (3.4)	3 (3.4)	0	0
**Other antivirals**	4 (4.5)	6 (6.8)	0	0
Favipiravir	4 (4.5)	5 (5.7)	0	0

Data are presented as n (%).

ACA, acalabrutinib; BSC, best supportive care; RoW, rest of the world.

### Primary endpoint

**Figure 2.**
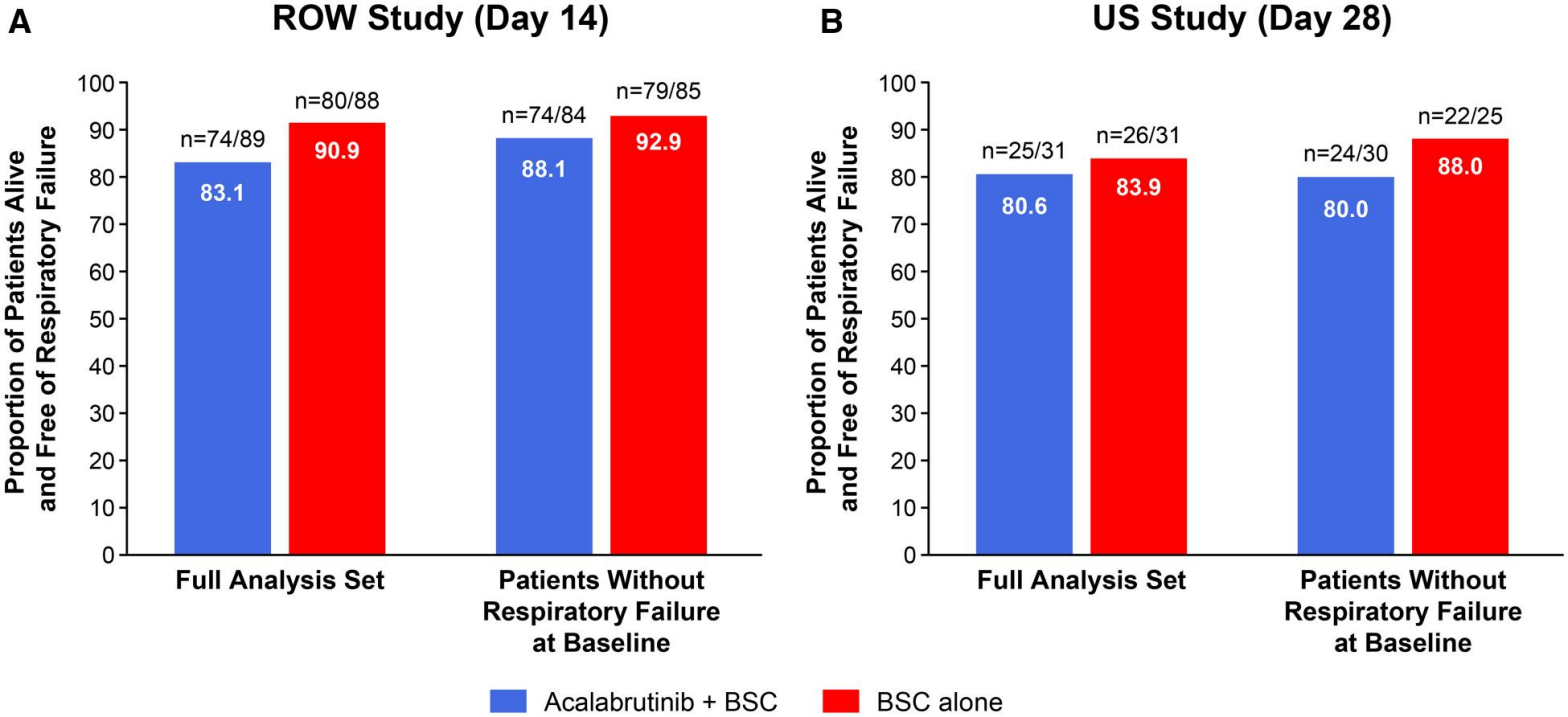
Primary endpoint: proportion of patients alive and free of respiratory failure in (A) the RoW study and (B) the US study. BSC, best supportive care; RoW, rest of the world; US, United States.

In both studies, the percentage of patients alive and free of respiratory failure at day 14 (RoW study) and day 28 (US study) was similar in the 2 treatment arms, and the between-arm differences were not statistically significant (Fig. 2, Table 3). A sensitivity analysis in the subgroup of patients without respiratory failure at baseline demonstrated similar results.

**Table 3.** Primary and key secondary endpoint results.

Endpoints	RoW study		US study	
	ACA + BSC	BSC alone	ACA + BSC	BSC alone
**Primary endpoints**				
**Full analysis set, n**	89	88	31	31
Proportion of patients alive and free of respiratory failure at day 14, n (%)	74 (83.1)	80 (90.9)	–	–
**Between-group difference (ACA + BSC—BSC), % (95% CI)**	**−7.8 (−18.7, 3.2)**		–	
Proportion of patients alive and free of respiratory failure at day 28, n (%)	–	–	25 (80.6)	26 (83.9)
**Between-group difference (ACA + BSC—BSC), % (90% CI)**	–		**−3.2 (−22.4, 15.9)**	
**Patients without respiratory failure at baseline (sensitivity analysis), n**	84	85	30	25
Proportion of patients alive and free of respiratory failure at day 14, n (%)	74 (88.1)	79 (92.9)	–	–
**Between-group difference (ACA + BSC—BSC), % (95% CI)**	**−4.8 (−14.8, 5.1)**		–	
Proportion of patients alive and free of respiratory failure at day 28, n (%)	–	–	24 (80.0)	22 (88.0)
**Between-group difference (ACA + BSC—BSC), % (90% CI)**	–		**−8.0 (−27.7, 11.7)**	
**Secondary endpoints**				
**Full analysis set, n**	89	88	31	31
Proportion of patients alive and free of respiratory failure at day 14, n (%)	–	–	25 (80.6)	27 (87.1)
**Between-group difference (ACA + BSC—BSC), % (95% CI)**	–		**−6.5 (−25.0, 12.1)**	
Proportion of patients alive and free of respiratory failure at day 28, n (%)	75 (84.3)	78 (88.6)	–	–
**Between-group difference (ACA + BSC—BSC), % (90% CI)**	**−4.4 (−15.6, 6.8)**		–	
Median (95/90% CI^a^) time to clinical improvement of ≥2 points, days	10 (8–12)	10 (8–11)	6 (5–7)	7 (5–11)
Median (95/90% CI^a^) time to SpO_2_ >94% on room air, days	9 (8–10)	9 (7–12)	14 (13–16)	NC (14–NC)
Proportion of patients with SpO_2_ >94% on room air by day 14, % (95/90% CI^a^)	68 (58–78)	70 (60–79)	50 (36–67)	38 (25–55)
Proportion of patients with SpO_2_ >94% on room air by day 28, % (95/90% CI^a^)	77 (67–85)	80 (71–88)	73 (59–86)	49 (35–65)
**Between-group difference (ACA + BSC—BSC), % (95% CI)**	**1.0 (0.7, 1.4)**		–	
**Between-group difference (ACA + BSC—BSC), % (90% CI)**	–		**1.8 (1.0, 3.3)**	

a 95% CI for the RoW study, and 90% CI for the US study.

ACA, acalabrutinib; BSC, best supportive care; CI, confidence interval; NC, not calculable; RoW, rest of the world; SpO_2_, oxygen saturation.

### Key secondary endpoints

In both studies, the percentage of patients alive and free of respiratory failure at day 28 (RoW study) and day 14 (US study) were similar to the results observed at day 14 and day 28 for the primary analysis; the proportion of patients was similar in the 2 treatment arms, and the between-arm differences were not statistically significant (Table 3).

Median time to clinical improvement of at least 2 points was also similar in the 2 treatment arms in both studies (Table 3). The time to an SpO_2_ of >94% on room air was similar between treatment arms in the RoW study; however, in the US study, SpO_2_ of >94% on room air in the BSC arm could not be determined (Table 3). In the RoW study, the proportion of patients with an SpO_2_ of >94% on room air who received acalabrutinib + BSC vs BSC alone was 68% and 70%, respectively, at day 14, and 77% and 80%, respectively, at day 28 (HR: 1.0, 95% CI: 0.7, 1.4; Table 3). In the US study, the proportion of patients with an SpO_2_ of >94% on room air who received acalabrutinib + BSC vs BSC alone was 50% and 38%, respectively, at day 14, and 73% and 49%, respectively, at day 28 (HR: 1.8, 90% CI: 1.0, 3.3; Table 3).

### Safety

In both studies, the incidence of AEs leading to discontinuation of acalabrutinib was low (RoW study: 5 [5.8%] patients; US study: 4 [13.3%] patients). Respiratory failure was the only AE that led to discontinuation in >1 patient in the RoW study, while there were no AEs that led to discontinuation in >1 patient in the US study.

In the RoW study, the most common AEs (occurring in ≥4 patients) in the acalabrutinib + BSC arm versus the BSC arm were headache (n = 10 [11.6%] vs n = 2 [2.2%]) and asthenia (n = 4 [4.4%] vs n = 0) (Table 4). In the US study, the most common AE (occurring in ≥4 patients) in the acalabrutinib + BSC arm versus the BSC arm was headache (n = 4 [13.3%] vs n = 0).

**Table 4.** Adverse events occurring in *≥*2 patients in either treatment arm.

**SOC, MedDRA PT** ^a^	**Number (%) of patients** ^b^			
	RoW study		US study	
	ACA + BSC (n = 86)	BSC alone (n = 91)	ACA + BSC (n = 30)	BSC alone (n = 32)
**Patients with any AE**	**43 (50.0)**	**37 (40.7)**	**17 (56.7)**	**15 (46.9)**
**Infections and infestations**	**12 (14.0)**	**6 (6.6)**	**5 (16.7)**	**5 (15.6)**
Pneumonia	3 (3.5)	0	NR	NR
Pneumonia bacterial	0	2 (2.2)	NR	NR
Device-related infection	2 (2.3)	0	NR	NR
Urinary tract infection	1 (1.2)	1 (1.1)	0	2 (6.3)
**Nervous system disorders**	**12 (14.0)**	**4 (4.4)**	**4 (13.3)**	**2 (6.3)**
Headache	10 (11.6)	2 (2.2)	4 (13.3)	0
Paresthesia	2 (2.3)	0	0	1 (3.1)
**General disorders and administration site conditions**	**8 (9.3)**	**4 (4.4)**	**1 (3.3)**	**3 (9.4)**
Asthenia	4 (4.7)	0	NR	NR
Fatigue	2 (2.3)	0	NR	NR
Non-cardiac chest pain	2 (2.3)	0	NR	NR
Pyrexia	0	3 (3.3)	NR	NR
Chest pain	NR	NR	0	2 (6.3)
**Cardiac disorders**	**7 (8.1)**	**3 (3.3)**	**1 (3.3)**	**2 (6.3)**
Palpitations	3 (3.5)	0	NR	NR
**Gastrointestinal disorders**	**7 (8.1)**	**12 (13.2)**	**4 (13.3)**	**5 (15.6)**
Constipation	2 (2.3)	3 (3.3)	0	2 (6.3)
Diarrhea	2 (2.3)	5 (5.5)	NR	NR
Nausea	2 (2.3)	2 (2.2)	0	1 (3.1)
Vomiting	0	2 (2.2)	NR	NR
**Respiratory, thoracic, and mediastinal disorders**	**7 (8.1)**	**10 (11.0)**	**2 (6.7)**	**3 (9.4)**
Epistaxis	2 (2.3)	0	NR	NR
Respiratory failure	2 (2.3)	0	0	1 (3.1)
Cough	1 (1.2)	2 (2.2)	NR	NR
Pulmonary embolism	1 (1.2)	2 (2.2)	0	1 (3.1)
Dyspnea	0	2 (2.2)	NR	NR
**Metabolism and nutrition disorders**	**6 (7.0)**	**7 (7.7)**	**3 (10.0)**	**3 (9.4)**
Hypokalemia	3 (3.5)	1 (1.1)	NR	NR
Decreased appetite	2 (2.3)	1 (1.1)	NR	NR
Hypophosphatemia	1 (1.2)	2 (2.2)	NR	NR
Hypernatremia	0	2 (2.2)	NR	NR
Hyperglycemia	1 (1.2)	1 (1.1)	0	3 (9.4)
**Investigations**	**5 (5.8)**	**5 (5.5)**	**4 (13.3)**	**3 (9.4)**
ALT increased	1 (1.2)	1 (1.1)	3 (10.0)	0
AST increased	0	1 (1.1)	2 (6.7)	0
Transaminase increased	3 (3.5)	1 (1.1)	NR	NR
**Musculoskeletal and connective tissue disorders**	**5 (5.8)**	**3 (3.3)**	**1 (3.3)**	**0**
Back pain	2 (2.3)	1 (1.1)	NR	NR
**Psychiatric disorders**	**5 (5.8)**	**4 (4.4)**	**2 (6.7)**	**4 (12.5)**
Insomnia	3 (3.5)	3 (3.3)	1 (3.3)	3 (9.4)
Agitation	NR	NR	0	2 (6.3)
**Renal and urinary disorders**	**4 (4.7)**	**5 (5.5)**	**1 (3.3)**	**0**
Acute kidney injury	3 (3.5)	2 (2.2)	NR	NR
Urinary retention	2 (2.3)	0	NR	NR
Renal failure	1 (1.2)	2 (2.2)	NR	NR
**Vascular disorders**	**4 (4.7)**	**6 (6.6)**	**1 (3.3)**	**2 (6.3)**
Hypotension	2 (2.3)	0	0	1 (3.1)
Hypertension	1 (1.2)	4 (4.4)	1 (3.3)	0
**Blood and lymphatic system disorders**	**3 (3.5)**	**5 (5.5)**	**3 (10.0)**	**1 (3.1)**
Thrombocytopenia	3 (3.5)	0	NR	NR
Anemia	2 (2.3)	3 (3.3)	1 (3.3)	1 (3.1)
Leukocytosis	0	1 (1.1)	2 (6.7)	0
**Skin and subcutaneous tissue disorders**	**2 (2.3)**	**1 (1.1)**	**3 (10.0)**	**2 (6.3)**
Rash	1 (1.2)	0	2 (6.7)	1 (3.1)

a Patients with multiple events in the same PT are counted only once in that PT. Patients with events in >1 PT are counted once in each of those PTs.

b Percentages are based on the total numbers of patients in each treatment arm.

Includes TEAEs. For the ACA + BSC arm, this was defined as AEs starting or ongoing, AEs worsening after the first dose of study treatment, and AEs with start date up to the last dose of study treatment plus 28 (+3) days. For the BSC arm, this was defined as AEs starting or ongoing, AEs worsening after the date of randomization, and AEs with a start date up to 38 (+3) days after randomization.

ACA, acalabrutinib; AE, adverse event; BSC, best supportive care; MedDRA, Medical Dictionary for Regulatory Activities; NR, not reported; PT, preferred term; RoW, rest of the world; SOC, system organ class; TEAE, treatment-emergent adverse event.

Treatment-related AEs were rare. In both studies, the only AE considered to be related to acalabrutinib treatment that occurred in ≥1 patient was headache (RoW study: n = 2 [2.3%]; US study: n = 2 [6.7%]). In the RoW study, the most common AEs of grade ≥3 in the acalabrutinib + BSC arm were hypotension and respiratory failure (n = 2 [2.3%] each), while the most common grade ≥3 AEs occurring in the BSC arm were bacterial pneumonia and hypernatremia (n = 2 [2.2%] each). No grade ≥3 AEs occurred in more than 2 patients in either treatment arm. Furthermore, no grade ≥3 AEs occurred in more than 1 patient in either treatment arm in the US study.

In the RoW study, SAEs were reported in 7 (8.1%) patients in the acalabrutinib + BSC arm and in 2 (2.2%) patients in the BSC arm. In the US study, SAEs were reported in 4 (13.3%) patients in the acalabrutinib + BSC arm and in 6 (18.8%) patients in the BSC arm. The only SAEs reported in more than 1 patient in either treatment arm of the RoW study were pneumonia (acalabrutinib + BSC: n = 2 [2.3%]; BSC alone: n = 0) and respiratory failure (acalabrutinib + BSC: n = 2 [2.3%]; BSC: n = 0). In the US study, the only SAE reported in more than 1 patient in either treatment arm was urinary tract infection (acalabrutinib + BSC: n = 0; BSC: n = 2 [6.3%]). In total, 17 patients died during the RoW study (acalabrutinib + BSC: n = 8 [9.0%]; BSC: n = 9 [10.2%]), and 4 patients died during the US study (acalabrutinib + BSC: n = 2 [6.5%]; BSC: n = 2 [6.5%]). Death due to COVID-19 only was reported in 11 patients in the RoW study and in 3 patients in the US study. In the RoW study, death due to both AEs and the underlying disease occurred in 3 patients in the acalabrutinib + BSC arm (cardiac arrest, respiratory failure, and sepsis), and in 2 patients in the BSC arm (septic shock and chronic obstructive pulmonary disease); death due to AE only was reported in 1 additional patient in the acalabrutinib + BSC arm (respiratory failure). No fatal AE reported as the primary cause of death was considered causally related to acalabrutinib.

### Biomarker analysis

**Figure 3.**
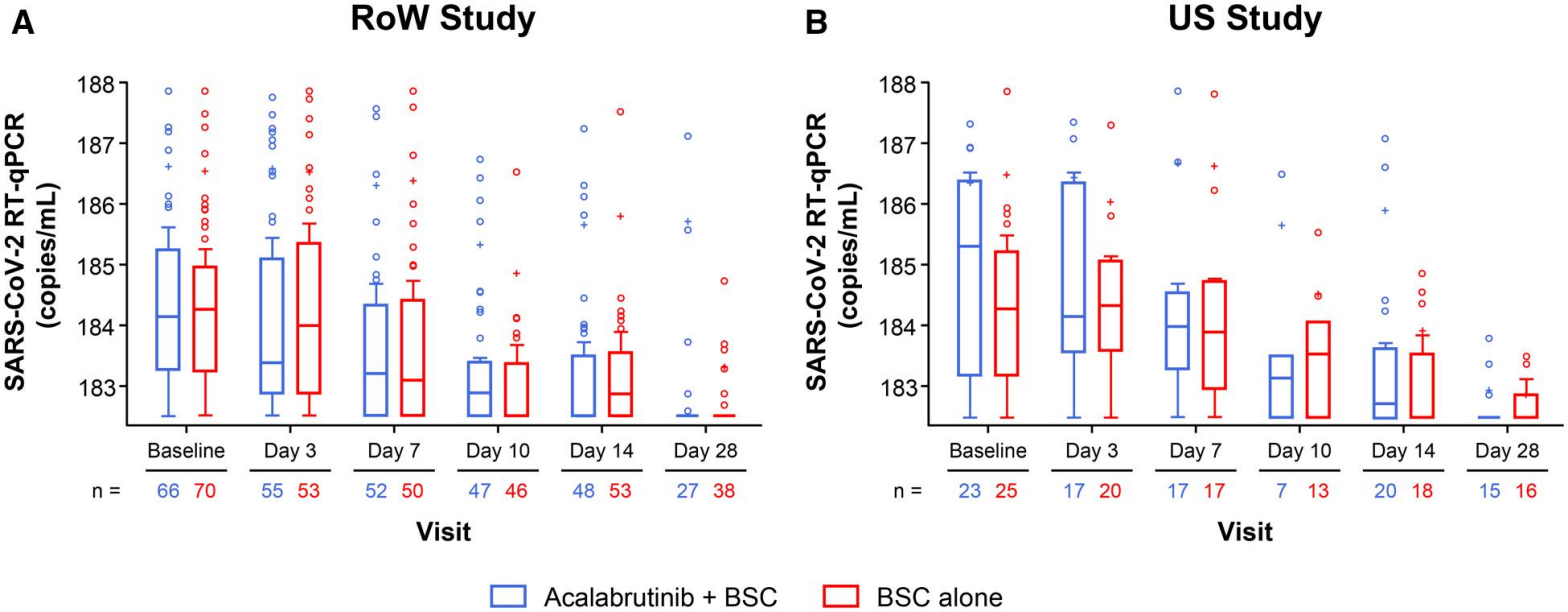
SARS-CoV-2 viral load profile over time in (A) the RoW study and (B) the US study. BSC, best supportive care; RoW, rest of the world; RT-qPCR, real-time quantitative polymerase chain reaction; SARS-CoV-2, severe acute respiratory syndrome coronavirus 2; US, United States.

**Figure 4.**
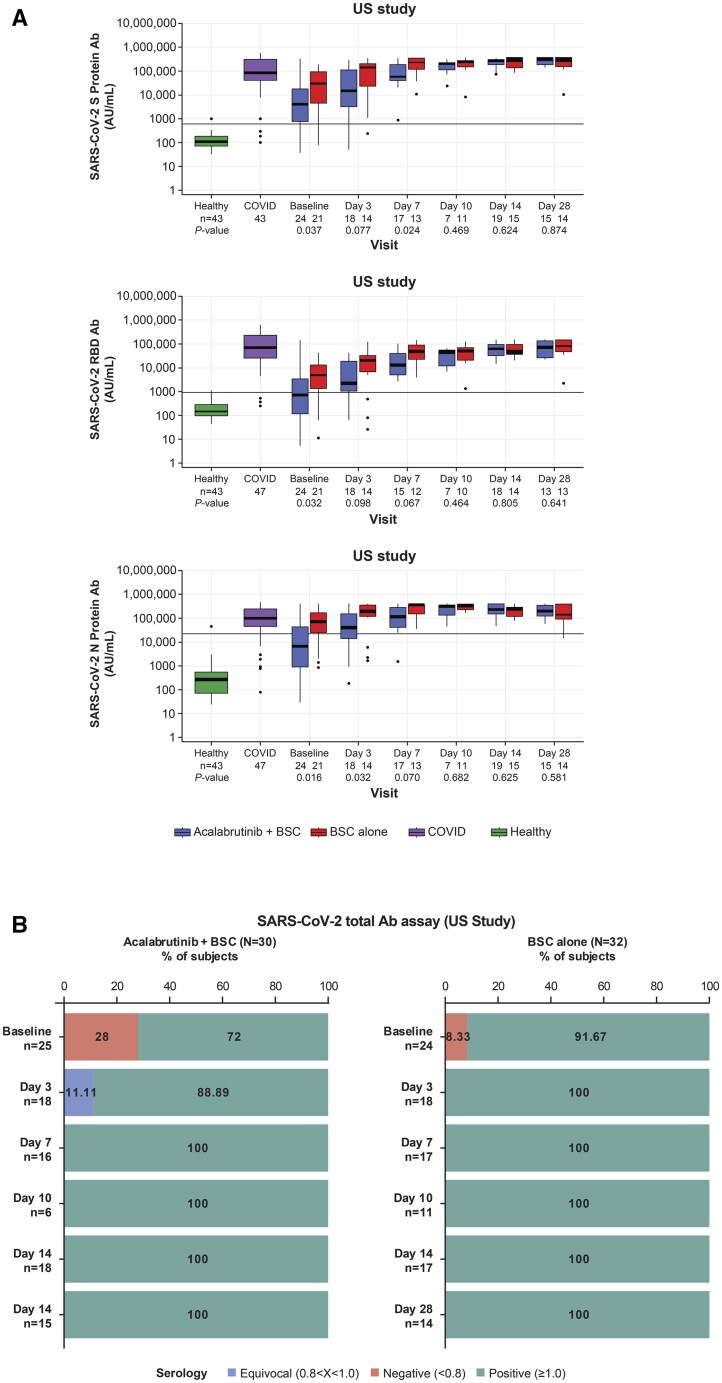
SARS-CoV-2 antibody detection in the US study. (A) Detection of antibodies against the SARS-CoV-2 spike, RBD, and nucleocapsid proteins, and (B) detection of total SARS-CoV-2 antibodies. Ab, antibody; BSC, best supportive care; COVID, coronavirus disease 19; N, nucleocapsid; Quant, quantification; RBD, receptor-binding domain; S, spike; SARS-CoV-2, severe acute respiratory syndrome coronavirus 2; US, United States.

Viral load decreased over time in all treatment arms from both studies (Fig. 3). Although the addition of acalabrutinib to BSC did not improve the clearance of SARS-CoV-2 virus relative to patients receiving BSC alone in the RoW study, the clearance of SARS-CoV-2 virus seemed to be faster with acalabrutinib + BSC compared with BSC in the US study. In the US study, the BSC arm had higher levels of SARS-CoV-2 antibodies compared with patients in the acalabrutinib + BSC arm at study enrollment through day 7 of treatment (Fig. 4). Treatment with acalabrutinib did not prevent the generation of SARS-CoV-2 antibodies.

**Figure 5.**
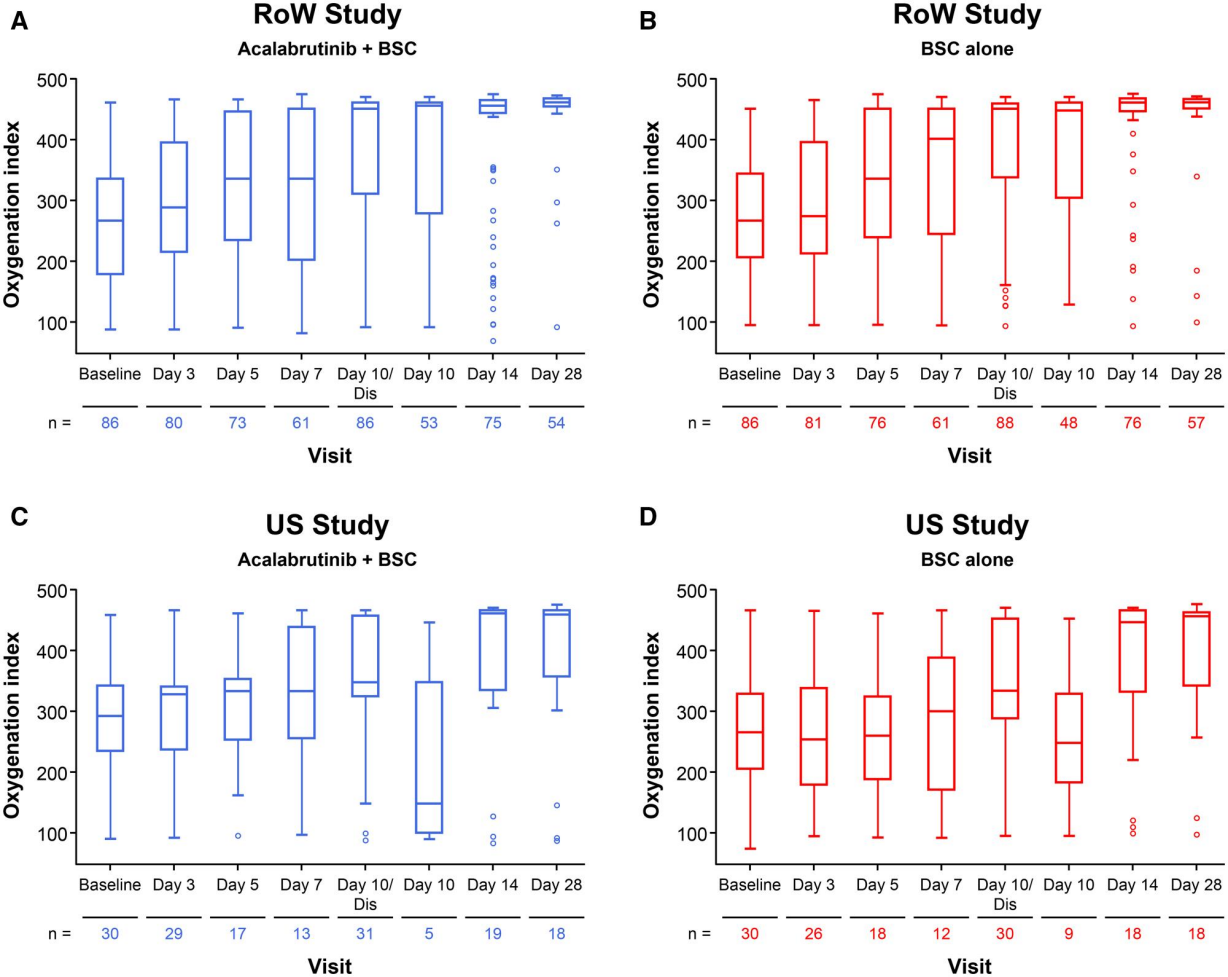
Oxygenation index over time in for (A) acalabrutinib + BSC in the RoW study, (B) BSC alone in the RoW study, (C) acalabrutinib + BSC in the US study, and (D) BSC alone in the US study. Oxygenation index = SpO_2_/FiO_2_, where SpO_2_ is a percentage and FiO_2_ is a decimal. For patients discharged from hospital prior to day 10, this is the last post-baseline assessment reported. For patients discharged from the hospital on or after day 10, this is the day 10 assessment. BSC, best supportive care; Dis, discontinuation; RoW, rest of the world; US, United States.

**Figure 6.**
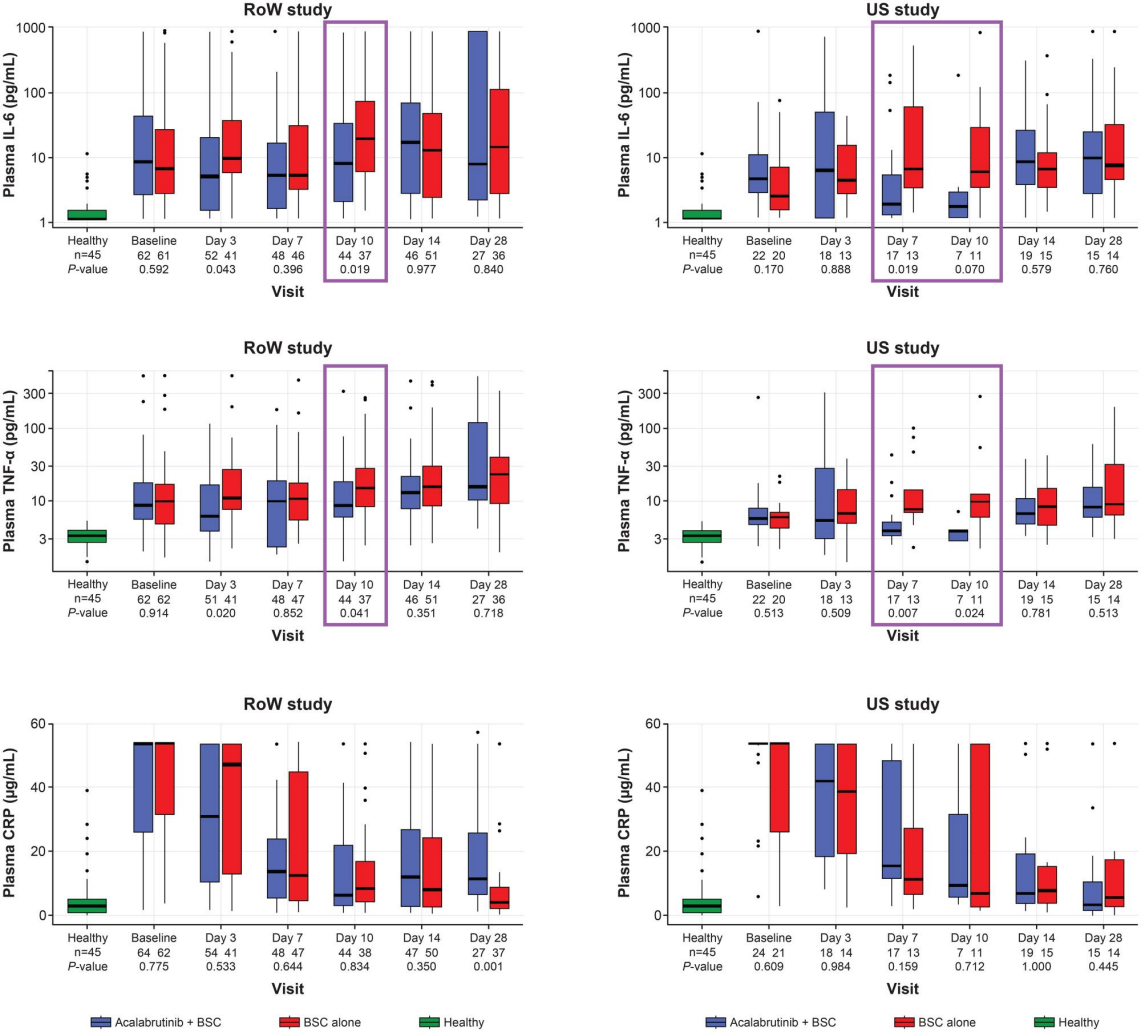
Levels of inflammatory cytokines over time. BSC, best supportive care; CRP, C-reactive protein; IL-6, interleukin 6; RoW, rest of the world; TNF-α, tumor necrosis factor-alpha; US, United States.

The oxygenation index (SpO_2_/fraction of inspired oxygen) generally increased over time in both studies, irrespective of the treatment received (Fig. 5). Absolute lymphocyte count generally increased over time in both studies (Table S1). Acute-phase protein serum amyloid A was elevated in patients with COVID-19 in all treatment arms in both studies but returned to normal levels as the viral load decreased (Fig. S1). Similarly, inflammatory cytokines including IL-6, tumor necrosis factor-alpha (TNF-α), and CRP were elevated in patients with COVID-19 at baseline compared with levels in healthy controls in both the RoW and US studies. In both studies, patients receiving acalabrutinib + BSC had lower levels of both IL-6 and TNF-α compared with patients receiving BSC at day 10 (Fig. 6). Chemokine analysis demonstrated that treatment with acalabrutinib dampened production of the BTK-dependent chemokines MIP-1α (US study only), MIP-1β, thymus- and activation-regulated chemokine (TARC/CCL17) (US study only), and MCP-1 (Fig. S2). In the US study, ordinal scale clinical improvement scores support modulation of the inflammatory response by acalabrutinib (Fig. S3). Overall, despite these observed variations in chemokines and cytokines in both cohorts, none correlated with any meaningful clinical outcome.

## Discussion

Data from the 2 studies presented herein show that even though treatment with acalabrutinib plus BSC dampened production of certain inflammatory cytokines, the addition of acalabrutinib did not improve clinical outcomes in patients with severe COVID-19 in terms of survival and respiratory failure at days 14 and 28 compared with BSC alone. All patients in the RoW study had serological conversion by day 1. The addition of acalabrutinib to BSC did not improve the clearance of the SARS-CoV-2 virus; however, acalabrutinib did not negatively impact SARS-CoV-2 antibody production, which was similar across treatment groups. Although trends were observed in the levels of cytokines measured, the impact of acalabrutinib on cytokine production was not marked and did not correlate with better outcomes. Importantly, changes in the plasma concentration of BTK-mediated chemokines TARC/CC17 and MCP-1 were observed, demonstrating that acalabrutinib did engage “on target” BTK as expected. BTK inhibition may have had less of an impact on cytokine signaling due to alternative mechanisms that are not BTK-mediated or the association with other BSC treatments such as corticosteroids. Although acalabrutinib in combination with BSC was not shown to improve survival and respiratory failure outcomes in patients with severe COVID-19 compared with BSC alone, future studies could identify patient groups that may benefit from this combination, particularly those on supplemental oxygen who are not in respiratory failure. Additionally, the clinical improvements measured by ordinal scale reported in the US study may reflect acalabrutinib’s impact in modulating the inflammatory response by reducing the plasma levels of BTK-mediated cytokines; however, further research is warranted to confirm this hypothesis.

The use of BTK inhibitors in patients with COVID-19 has previously been reported.^8^^,^^23^ Furthermore, use of acalabrutinib in patients hospitalized with severe COVID-19 showed improved oxygenation in the majority of patients, normalization of inflammatory cytokine levels, and improvement in lymphopenia.^8^ Moreover, a systematic review of outcomes from studies investigating the use of BTK inhibitors in patients with COVID-19 identified trends for decreased oxygenation requirements and/or decreased hospitalization and the need for care, but noted that these results were based on a small number of studies of mostly small sample sizes, and as such, should be interpreted with caution.^24^

The results of the present study may have been affected by baseline demographics and disease characteristics, including a higher BMI in the US population compared with the BMI in the RoW population, especially since obesity is associated with more severe COVID-19 symptoms and a worse prognosis, as well as reduced lung function and a poor response to mechanical ventilation.^25^ The results of the present study are also likely to have been impacted by the types of BSC administered, which included the common use of corticosteroids and antivirals, but also other therapies in a rapidly evolving treatment landscape. Most patients received corticosteroids compared with the lower numbers of patients who received other types of treatment. The use of such treatments during the enrollment period for these studies may have led to a substantial reduction in the mortality and morbidity in patients hospitalized with COVID-19, which, in turn, minimized the impact that additional treatment regimens can have on patient prognosis and recovery. Data from a systematic review with meta-analysis confirmed that the mortality was lower in patients with severe COVID-19 who received corticosteroids at any time during treatment than those who did not receive any corticosteroids (odds ratio [OR] 0.70; 95% CI: 0.54–0.92) and that the early use of corticosteroids in these patients was even more impactful (OR 0.37; 95% CI: 0.25–0.57).^26^ Patients who could not swallow pills or who, in the opinion of the treating physician, were likely to require mechanical ventilation within 24 h of screening were excluded and patients who could no longer swallow were ineligible to continue acalabrutinib, which limits the interpretation of the results to patients capable of taking acalabrutinib.

Efforts were made in the protocol design to minimize the impact of the above-mentioned potential confounders; however, the unique spread of SARS-CoV-2 and the available approved therapies in different countries could not be addressed in real time given the accelerated pace of the evolving pandemic. Additionally, it is difficult to draw conclusions based on the use of specific medications that were administered to small numbers of patients in these studies. Furthermore, prior studies indicated that administration of BTKis, including ibrutinib and acalabrutinib, may increase the risk of bacterial and/or fungal infections.^27^ Despite the differences in dose and duration, and the potential for increased risk of infections, the overall safety profile of acalabrutinib reported in this study of patients with COVID-19 who required hospitalization was consistent with that reported in prior studies in patients with B-cell malignancies.^28–30^ Further research on the use of immune-modulating therapies in patients with SARS-CoV-2, such as dexamethasone, tocilizumab, and baricitinib, may be warranted to inform best practices in treating COVID-19.^12^

In conclusion, these studies found biological activity but no clinical benefit of adding acalabrutinib to BSC in patients hospitalized with COVID-19. The frequent use of corticosteroids and antiviral drugs in patients with COVID-19 poses a challenge to demonstrating clinical benefit with acalabrutinib administration.

## Supplementary Material

vlaf023_Supplementary_Data

## Data Availability

Data underlying the findings described in this manuscript may be obtained in accordance with AstraZeneca’s data sharing policy described at https://astrazenecagrouptrials.pharmacm.com/ST/Submission/Disclosure. Data for studies directly listed on Vivli can be requested through Vivli at www.vivli.org. Data for studies not listed on Vivli can be requested through Vivli at https://vivli.org/members/enquiries-about-studies-not-listed-on-the-vivli-platform/. AstraZeneca Vivli member page is also available outlining further details: https://vivli.org/ourmember/astrazeneca/.
