# Supplementary material for: Efficacy and safety of acalabrutinib with best supportive care versus best supportive care in patients with COVID-19 requiring hospitalization
Source: Immunohorizons. 2025 Jun 3;9(7):vlaf023. doi: 10.1093/immhor/vlaf023 (PMC12133263; doi:10.1093/immhor/vlaf023)
Supplement: vlaf023_Supplementary_Data [file vlaf023_supplementary_data.pdf]

Supplemental Materials

Table S1. Absolute lymphocyte counts and change from baseline over time

	RoW study		US study	
	**ACA + BSC (n=89)**	**BSC alone (n=88)**	**ACA + BSC (n=31)**	**BSC alone (n=31)**
**Absolute lymphocyte counts (x10^9/L), mean (SD)**				
Baseline	1.15 (0.58)	1.16 (0.66)	0.80 (0.42)	1.07 (0.61)
Day 3	1.36 (0.85)	1.39 (0.79)	1.23 (0.94)	1.24 (0.90)
Day 5	1.61 (0.93)	1.76 (1.23)	1.05 (0.92)	1.38 (0.81)
Day 7	1.43 (0.88)	1.67 (0.95)	1.34 (1.19)	1.47 (1.06)
Day 10/discontinuation	1.87 (1.00)	1.84 (0.91)	1.41 (0.86)	1.79 (1.06)
Day 10	1.71 (1.01)	1.73 (0.79)	0.80 (0.10)	1.90 (1.55)
Day 14	1.68 (0.78)	1.86 (0.85)	1.46 (0.55)	1.72 (0.63)
Day 28	1.88 (0.81)	1.91 (0.61)	1.73 (0.43)	1.77 (0.70)
**Mean percentage change from baseline**				
Baseline	–	–	–	–
Day 3	31.7	36.8	54.8	34.7
Day 5	55.8	87.3	28.2	76.9
Day 7	51.7	79.3	46.2	102.8
Day 10/discontinuation	73.3	99.7	100.8	97.5
Day 10	98.6	83.1	67.4	118.3
Day 14	74.7	91.6	108.6	106.9
Day 28	89.4	96.6	135.3	127.1

ACA, acalabrutinib; BSC, best supportive care; RoW, rest of the world; SD, standard deviation; US, United States.

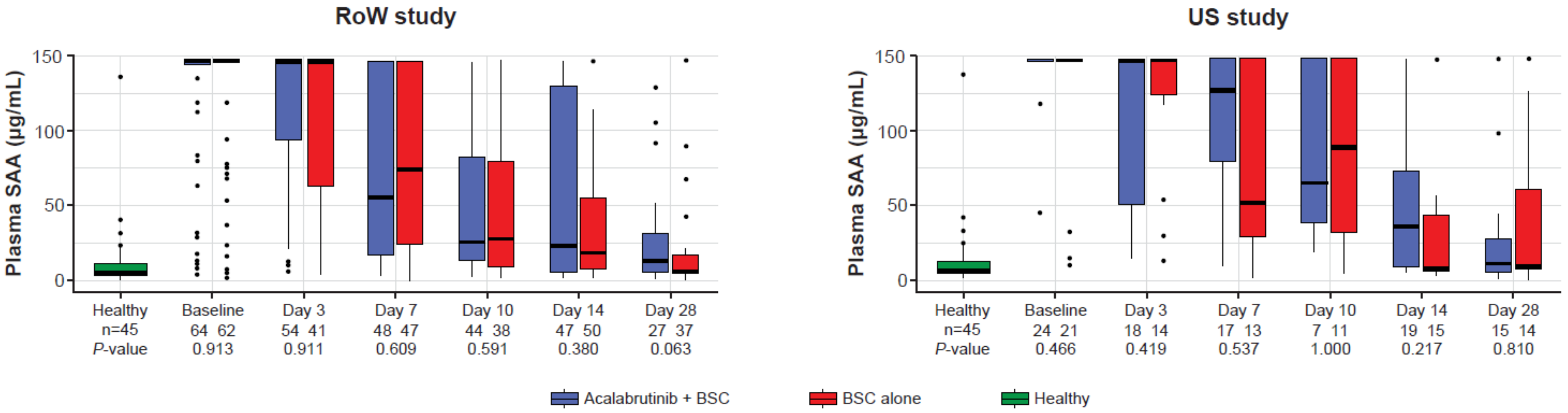

Figure S1. Acute phase protein over time: serum amyloid A. BSC, best supportive care; RoW, rest of the world; SAA, serum amyloid A; US, United States.

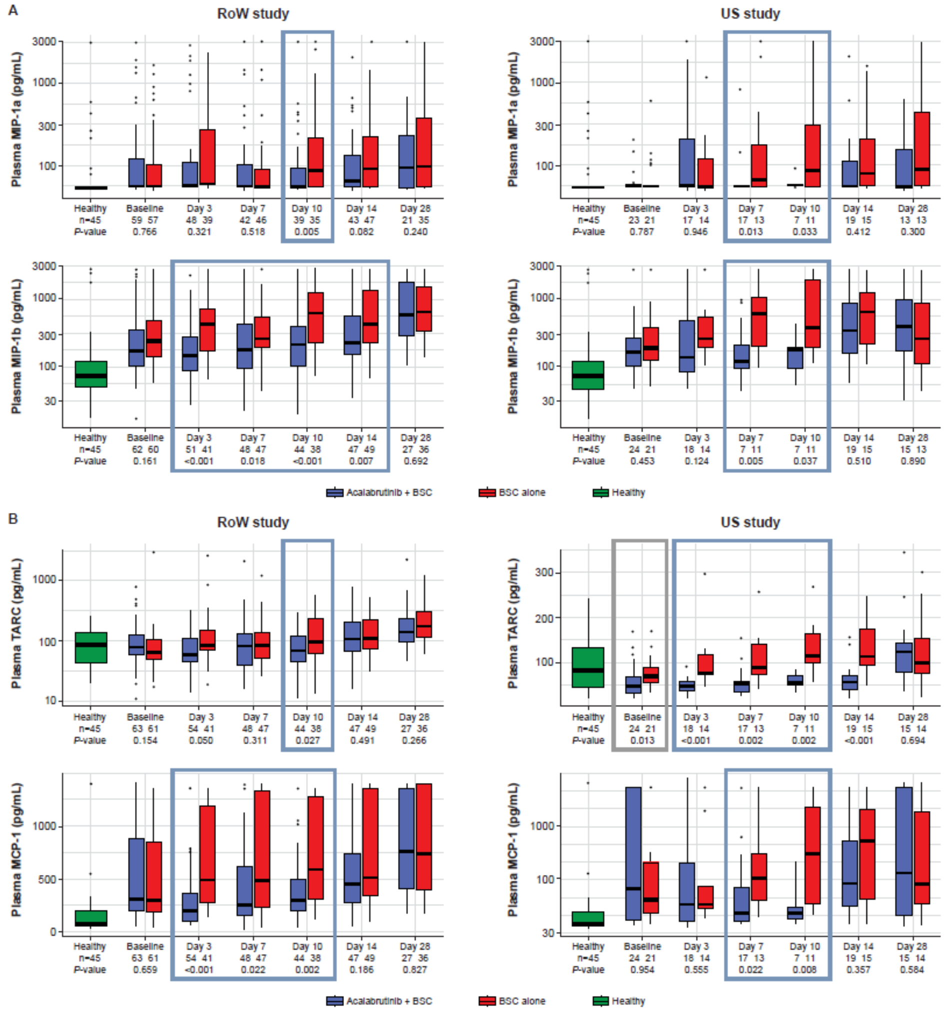

Figure S2. Levels of inflammatory chemokines over time for **A.** MIP-1α and MIP-1β and **B.** TARC (CCL17) and MCP-1. BSC, best supportive care; MCP-1, monocyte chemoattractant protein-1; MIP-1α, macrophage inflammatory protein-1 alpha; MIP-1β, macrophage inflammatory protein-1 beta; RoW, rest of the world; TARC (CCL17), thymus- and activation-regulated chemokine; US, United States.

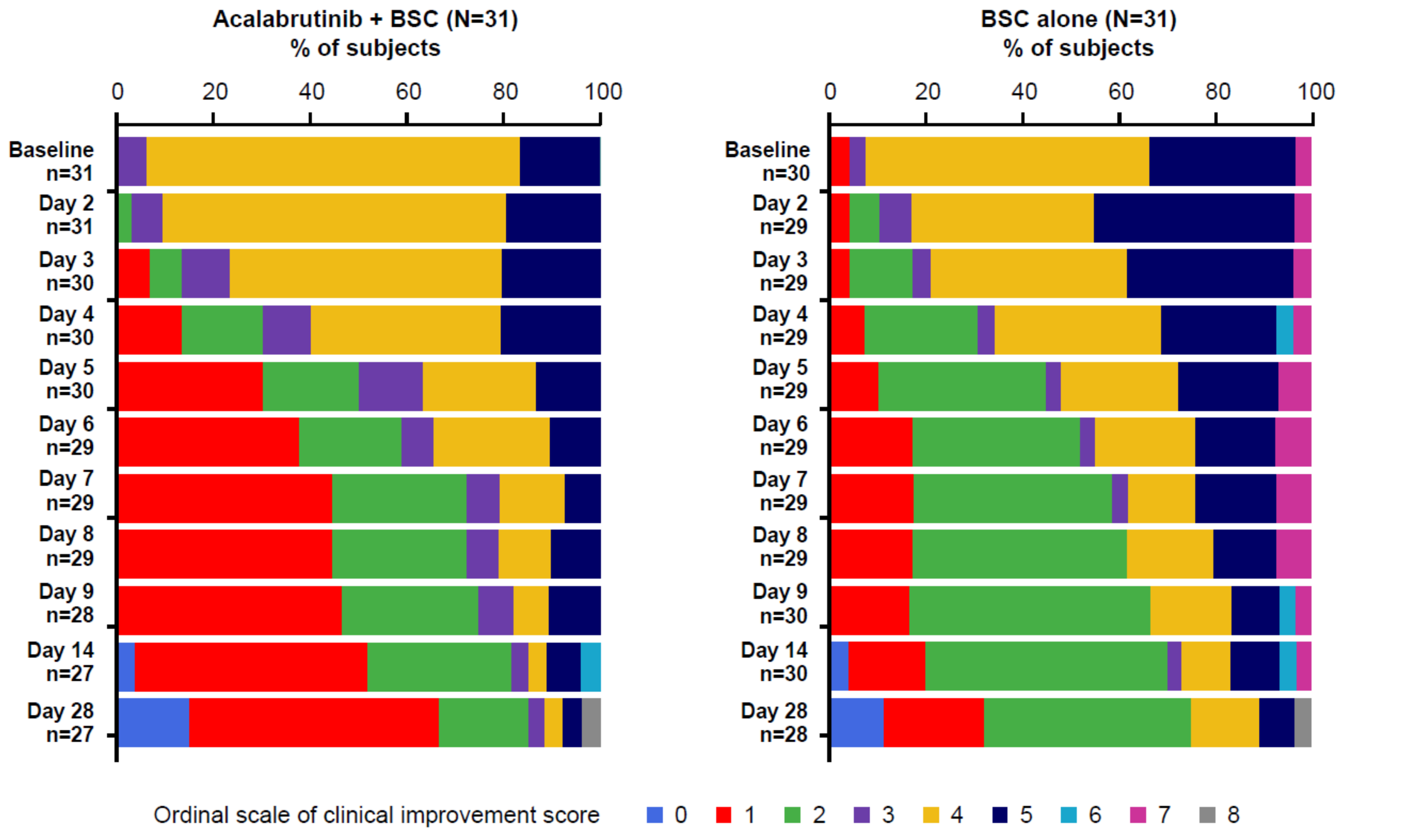

Figure S3. Physiological effects of treatment in ordinal scale of clinical improvement score (US study). BSC, best supportive care.
